# Statistical properties of simple random-effects models for genetic heritability^*^

**DOI:** 10.1214/17-EJS1386

**Published:** 2018-02-15

**Authors:** David Steinsaltz, Andrew Dahl, Kenneth W. Wachter

**Affiliations:** Department of Statistics, University of Oxford; Wellcome Trust Centre for Human Genetics and Department of Statistics, University of Oxford; Departments of Demography and Statistics, University of California

**Keywords:** Primary 92D10, secondary 62P10, 62F10, 60B20, heritability, random-effects models, random matrices, Marcenko–Pastur distribution, GCTA

## Abstract

Random-effects models are a popular tool for analysing total narrow-sense heritability for quantitative phenotypes, on the basis of large-scale SNP data. Recently, there have been disputes over the validity of conclusions that may be drawn from such analysis. We derive some of the fundamental statistical properties of heritability estimates arising from these models, showing that the bias will generally be small. We show that that the score function may be manipulated into a form that facilitates intelligible interpretations of the results. We go on to use this score function to explore the behavior of the model when certain key assumptions of the model are not satisfied — shared environment, measurement error, and genetic effects that are confined to a small subset of sites.

The variance and bias depend crucially on the variance of certain functionals of the singular values of the genotype matrix. A useful baseline is the singular value distribution associated with genotypes that are completely independent — that is, with no linkage and no relatedness — for a given number of individuals and sites. We calculate the corresponding variance and bias for this setting.

## 1. Introduction

Genome-Wide Complex-Trait Analysis, known as GCTA, introduced by Jian Yang and collaborators in 2010 in [51], has led both to a profusion of research findings across the biomedical and social sciences and to exuberant controversy. The general method, as distinct from the GCTA package of algorithms, is now widely known as GREML, for “Genomic Restricted Maximum Likelihood.” Use of the method has leapt ahead of clarity about its statistical properties. There has been extensive discussion of sensitivity to violation of assumptions, but no consensus on performance when its most basic assumptions are satisfied. Is statistical bias a concern even in the simplest settings? Some say yes [20]. Some say no [53]. Do tractable standard errors depend on the presence of residual population stratification? Formulas in the literature [46] leave the answer murky.

In this paper we seek to settle these basic questions.

- For “Simple GREML”, defined below, with *n* respondents and *p* genetic markers, we go beyond order $1/\sqrt{n}$, derive formulas for bias to order 1/*n*, and show this intrinsic bias to be negligible in practice.
- We present interpretable expansions for bias and standard error drawing on eigenvalue theory, depicting the contrasts between standard errors in the absence and in the presence of population stratification.
- For less simple settings, we consider known sources of bias including shared environment and measurment error, and characterize and bound bias arising from
  - observed causal genetic variants at a subset of sites atypical with respect to their linkage statistics, and
  - unobserved causal genetic variants.
- In a companion paper [44], we consider negative estimated values for the GREML parameter representing heritability which, we argue, remain meaningful within the model and should not be excluded.

The data for GREML are assays of very large numbers of Single Nucleotide Polymorphisms (SNPs) in the genomes of individuals along with measurements of a putatively heritable trait. The model that GREML fits via the technique of Restricted Maximum Likelihood is defined in Section 2. For simplicity, we refer to it as “the GREML model”. It is an example of a “linear mixed model,” in which the contribution of SNPs to trait values are treated as random effects. (We do not consider fixed effects in this paper, but the same arguments would apply to reduced phenotypes after elimination of fixed effects in a mixed model.) Alternative estimation methods for the model such as LD-score Regression and Haseman-Elston regression have also come into wide use, bringing up statistical issues paralleling those we examine here for GREML.

Mixed models form a natural framework for the estimation of total heritability for traits whose variability is determined by a wide variety of sites, rather than by specific identifiable SNPs that each have strong influence. GREML found notable application in the GCTA work by Yang and Visscher and associates to identify “missing heritability” in height [51, 49] and other complex traits, and many groups have followed their lead.

The goal of these methods is to estimate total additive heritability, without the overfitting that arises in attempts to identify specific loci influencing a trait. As we have said, most examination of pitfalls – including two papers [50, 55] with “pitfalls” in the title – have emphasized issues that arise from kinds of model misspecification: from single large-effect alleles (better treated as fixed effects [17, 38, 55]), from reliance on linkage disequilibrium when causal alleles themselves are unobserved [51, 41, 23, 42], from nonlinear increase in heritability estimates with increasing numbers of SNPs (reflecting saturation of coverage of a smaller subset of genuinely causal loci), and from ascertainment bias in binary traits [24, 4, 13].

Recently, Kumar *et al.* [20] have taken a different tack, criticising GREML on statistical grounds, on what might be considered issues of inherent mathematical fallibility for estimation in models relying on high-dimensional covariates. Their paper has elicited rebuttals from Yang *et al.* [53] and [54] and rejoinders to the rebuttal [21] and [19] from Kumar *et al*. Parts of that exchange are devoted to GREML and the GREML model in more complicated settings, but our results for “Simple GREML” in this paper settle some of the points in contention.

In “Simple GREML”, as we use the words, all causal SNPs are observed, all observed SNPs are causal, and the sizes of causal effects are all drawn independently from the same centered normal distribution. We abstract away from the need, important in practice, for tagging unobserved causal alleles by observed alleles by taking advantage of linkage disequilibrium. Non-genetic variance is contributed by independent draws for each sample from another centered normal distribution. Fundamental statistical properties are brought into the spotlight by studying this streamlined version.

Taking as a starting point our treatment of bias and variance in Simple GREML, to be described shortly, we go on in later sections of this paper to add our own perspective on model misspecification. It is a crucial motivation for our approach. In recent decades the foundations of statistics have turned away from consistency in settings where the the data are sampled from a “true model” to estimation in what B. Lindsay and J. Liu have termed a “model-false world” [26]. It is important to understand the behavior of model parameters — such as heritability in GREML – that form the basis for scientific discussion, when the data do not come from the model that gave them a precise meaning. Some attempts in this direction were made by [20], but these are not founded on a consistent theory of model misspecification and suffer from some mathematical misunderstandings, as we shall point out.

Unlike the setting in Simple GREML, in more complicated settings only a subset of all SNPs may have causal effects on the phenotype, sometimes observed SNPs, sometimes unobserved ones. With regard to this topic, Section 4, under the heading of “Model Misspecification”, assesses potential biases at several levels of complexity. Yang *et al.* remark at the outset of [51] that it is harmless to relax the assumptions of Simple GREML to allow non-zero effect sizes to be confined to an unknown random subset of observed SNPs. We agree with this claim up to a point, but show that it needs some correction. As Yang *et al.* and others [22] recognize, non-zero effects solely on a **fixed**, unidentified subset of SNPs can, in principle, introduce a bias of arbitrary size and direction. While it is true (as we confirm, using more direct arguments) that this bias averages out close to zero when the causal set is considered as a randomly chosen subset of all SNPs, this bias increases the expected error in the heritability estimates. Under strong but reasonable additional assumptions, however, we can estimate this additional error, and show that it is small under most circumstances. Non-zero effects on unobserved causal SNPs in linkage disequilibrium with observed SNPs is a complicated, widely-discussed issue, on which we offer some brief views of our own in Section 4.4.2.

Exactly this sort of misspecification forms the central subject of the recent work by Jiang *et al.* [16]. Those authors go into more mathematical detail than the present work and provide similar conclusions with regard to consistency as the matrix size goes to infinity. But they confine themselves to the special case of i.i.d. random genotype matrices. Our results, which complement theirs, offer both an interpretable description of the bias arising from particular genotype matrices and of the variance arising from averaging over random subsets of potential causal loci. Earlier work [15] also provides useful background on the theoretical underpinnings of Restricted Maximum Likelihood, although the approach and the orientation are substantially different from those adopted here.

We now return to Simple GREML. For us, the matrix of genotypes is fixed and observed without error; we condition on it. Statistical properties of GREML estimates depend on the matrix through its squared singular values, which are the eigenvalues of the Genetic Relatedness Matrix. Numbers of samples *n* are assumed to be in the tens or hundreds of thousands, and numbers of SNPs *p* much larger; the ratio *μ* of samples to SNPs is a key parameter.

Empirical studies have alluded to or reported a wide variety of patterns for sets of squared singular values, sometimes concentrated near unity, sometimes dispersed across orders of magnitude. We consider two extremes of contrasting settings intended to bracket the reported patterns in the literature. First, (A), is the “independent setting,” with a genotype matrix resembling one random draw from an ensemble of matrices with independent entries, thereby assuming an absence of population stratification and of linkage disequilibrium. Second, (B), is a “stratified setting”, represented in this paper by two flavors of stylized distributions, evoking genotype matrices whose singular values suggest deep population stratification.

While the independent setting is a frankly artificial idealization, it serves as an indispensable guide. Intuition suggests that the information content from a set of SNPs in linkage disequilibrium should resemble the information content from a smaller set of independent SNPs. The independent setting with a downward-adjusted ratio of SNPs to samples might be a good starting point for realistic genotype matrices. With this advantage in mind, we devote special attention to explicit formulas for estimator bias and variance in the independent setting

The key tool in our statistical analysis of GREML is a profile likelihood function that reduces the estimation problem to finding the root of a univariate function and simplifies simulations. The model is set out in Section 2 and the profile likelihood and bias formula are derived in Section 3 under the assumptions of Simple GREML. Section 4 takes up the more complicated issues associated with subsets of causal SNPs and other aspects of model specification. Section 5 derives the formulas relevant to the independent setting, and Section 6 sums up.

Our findings about bias and variance run contrary to conclusions of Kumar *et al.* in [19]. In particular, we see the limitations on accurate GREML estimation in the absence of stratification arising not from bias but from large standard errors, in contrast to their conclusions about the salience of bias. We see population stratification in small doses enhancing the accuracy of GREML estimates of heritability — at a cost in interpretability, as population structure typically correlates with environmental confounders [36, 2] — in contrast to their general contention that stratification undermines the stability of estimates. However, we do see large departures from the independent setting imperilling the accuracy of GREML, leading us to be less sanguine than Yang *et al*. [53] about the statistical properties of these genome-wide random effects models.

Many further issues about GREML arise in more complicated settings under more flexible assumptions and rightly engender continuing debate. However, given the impact of increasingly complex generalizations of Simple GREML to test association [18, 17, 59, 27, 38, 45, 35, 31, 30, 29, 60, 3], partition heritability [57, 52, 9], predict phenotype values [37, 58, 39, 6], and learn “co-heritabilities” [43, 25, 5, 7], it is important, if possible, to reach consensus on some core facts. Our analysis of bias and variance for Simple GREML aims at this goal.

## 2. The GREML model

We suppose we are given a data set consisting of an *n* × *p* matrix *Z*, considered to represent the genotypes of *n* individuals, measured at *p* different loci. There is a vector **y**, representing a scalar observation for each of the *n* individuals. The underlying observations are counts of alleles taking the values 0, 1, 2, but the genotype matrix is centered to have mean zero in each column and normalized to have mean square over the whole matrix equal to 1.

It is common practice to go further and normalize each column to have unit variance either empirically or under Hardy-Weinberg equilibrium. (SNPs far from Hardy-Weinberg equilibrium are generally excluded by quality control procedures, so these two alternatives amount to much the same thing.) Such normalization gives all columns equal weight in producing genetic effects. This assumption that normalized SNPs have i.i.d. effect sizes implies that unnormalized SNP effect sizes decrease with increasing allele frequency in a precise way [14, 48]. Except where noted, we do not assume this column-by-column normalization. However, the normalization of the sum of squares of the whole matrix is required for a sensible interpretation of the parameter representing heritability [41]. Moreover, heritability can be estimated under different assumed relationships between the allele frequency and SNP effect distribution [41], and non-standard assumptions yield different heritability estimates and may better model many real datasets [40].

The basic assumption of the model is the existence of a random vector **u** ∈ ℝ*^p^* of genetic influences from the individual SNPs such that

(1) y=Zu+ε.

The vectors **u** and *ε* are assumed to be independent and to have zero means and i.i.d. normal components. The variances are determined by two parameters, which are to be estimated: *θ* represents the precision (reciprocal variance) of the non-genetic noise and *ψ* represents the heritability, entering the model as the ratio of genetic variance to total variance. We use *ψ* rather than the more conventional *h*^2^ both because of the notational extravagance that results when these are raised to further powers, and also to accommodate possibly negative estimates, as is done in depth in [44]. It will be convenient at some points to use the parameter *ϕ* = *ψ*/(1 − *ψ*) in place of *ψ* itself. We allow the true values of the parameters to lie in the range *θ* > 0 and *ψ* ∈ [0, 1). Writing (*θ*_0_*, ψ*_0_) or (*θ*_0_*, ϕ*_0_) for the true values from which the data are generated, we have *u_j_* ~ 𝔑(0*, ϕ*_0_/(*pθ*_0_)) ~ 𝔑(0, *ψ*_0_/(*pθ*_0_(1 − *ψ*_0_))), and *ε_i_* ~ 𝔑(0, 1/*θ*_0_).

In the discussion by [20], much weight is placed on *Z* being a “random” matrix. There are several senses in which *Z* may reasonably be thought of as random:

1. The individuals are sampled from a larger population.
2. The SNPs have been selected from a larger set of possible SNPs.
3. The genotypes of individuals have been formed by random processes of mating, mutation, selection, and recombination.
4. There are random errors in the genotypes.

None of these substantially affects the analysis we carry through in this paper (although, with regard to the fourth kind, see Section 4.3). The model assumes that all genetic causality runs through *Z*, so that for purposes of estimation *Z* may simply be taken as a deterministic known quantity, a standard setup for covariates in regression models. On the other hand, as in standard linear regression models, some choices of independent covariates make the regression problem easier than others, so it is worth considering which *Z* may be likely to occur.

As we show shortly — and as [20] correctly point out — the properties of this statistical model are determined entirely by the singular-value spectrum of *Z*. In our independent setting (A), for a population without stratification and linkage disequlibrium, the empirical distribution of the singular values is expected to be close to a known limiting form, featured in Section 5 and depending only on the dimension ratio *μ* = *n*/*p*. In our stratified setting (B), with a proportion of singular values orders of magnitude larger than those typical of setting (A), qualitative generalizations need not rely on the detailed singular value spectrum.

## 3. The profile likelihood and bias formula

### 3.1. The likelihood function

We begin our derivation of expressions for bias and standard error in estimated heritability by reducing GREML likelihood estimation from a two-parameter to a one-parameter problem. We define a profile likelihood function whose local maximization only requires finding the zero of a univariate function. This device is the key to the derivation, and it offers the bonus of facilitating simulations.

We present terms for bias and variance up to order 1/*n*. Giving meaning to “order 1/*n*” requires an asymptotic framework. We could imagine the genotype matrix *Z* of fixed dimesions *n* and *p* to be imbedded in a sequence of matrices of increasing dimensions, typically for *n* increasing with *p*/*n* converging to a constant. Within the independent setting, such structure is easily specified; outside it, not so easily. Since the likelihood for *ψ* only depends on *Z* through the singular values, all we need is structure on a triangular array of singular values *s_n,i_* with *i* = 1, … *n*, typically just enough structure so that the empirical measures of the singular values for increasing *n* converge to a non-trivial limit.

We observe that the likelihood function is a sum of terms corresponding to the *s_n,i_*. The terms are independent but not identically distributed and themselves form a triangular array. Textbook theorems for maximum likelihood with independent observations do not, strictly speaking, cover the GREML model setup; our theorems (slightly) extend those theorems. As expected from the standard setup, we show that to order $1/\sqrt{n}$ estimator bias is zero. But $1/\sqrt{n}$ is not small enough, in many GREML applications, to make the next term, of order 1/*n*, negligible, if it comes with a large constant. We need to compute the next term explicitly, and do so in order to resolve conflicting claims about bias in the literature.

The expression for bias brings with it an expression for estimator variance to order 1/*n*, equivalent to the standard but unwieldy expression from Fisher Information, given, e.g., on pages 234–235 of [46], We go on to make bias and variance interpretable by expanding them in the independent setting in terms of dimension ratios and comparing with stylized cases for stratified settings.

Conditioned on *Z*, in terms of the Genetic Relatedness Matrix or GRM defined by *A* := *p*^−1^ZZ^*^, the measurements **y** are normally distributed with mean zero and covariance matrix

(2) $$C^{2}:=\theta_{0}^{-1}\;((\psi /(1-\psi ))\;A+I_{n}).$$

Let *Z* = *U* diag(*s_i_*)V^*^ be the singular-value decomposition of $Z/\sqrt{p}$, and rotate the observations to diagonalize the covariance matrix, obtaining

$$z:=U^{*}y.$$

The elements of **z** are independent centered normal random variables with variances

$\left(1-\psi +\psi s_{i}^{2})/(\theta (1-\psi )\right)$.

The log likelihood is then

(3) $$\ell (\theta ,\psi )=\frac{n}{2}\log\theta (1-\psi )-\frac{1}{2}\sum_{i=1}^{n}\log(1-\psi +\psi s_{i}^{2})-\frac{\theta }{2}\sum_{i=1}^{n}\frac{(1-\psi )z_{i}^{2}}{1-\psi +\psi s_{i}^{2}}.$$

Note that **z** depends only on *Z* and **y**, not on the parameters *θ* and *ψ*.

We observe here that [20] claim (without demonstration) that the presence of singular values in the denominator of the likelihood creates “instability” in estimates based on this likelihood when the singular values are small, and that the dependence on the projection onto left singular vectors creates instability when the singular values are close together. Neither is true. Their representation of the log likelihood differs from the one we have here by the addition of the log determinant of *A*. This is a very large number if there are singular values close to zero (and indeed infinite if singular values are zero), but the addition of a constant, however large, has no influence on likelihood-based estimation. (We note as well that the work done by Sylvester’s Theorem (their equation [A6]) is unnecessary as soon as we interpret the determinant as a product of squared singular values. Furthermore, in the case when a singular value is exactly zero, which occurs automatically when using de-meaned SNPs, the conditions for applying Sylvester’s Theorem are not satisfied.) Similarly, under the assumptions of the model, the *z_i_* are independent normal random variables, with variances $\theta^{-1}{\left(1-\psi \right)}^{-1}(1-\psi +\psi s_{i}^{2})$, which are positive so long as the environmental contribution *θ*^−1^ is positive.

### 3.2. MLE Bias and Variance

We define

$$w_{i}(\psi ):=\frac{1-\psi }{1-\psi +\psi s_{i}^{2}}$$

and

$$v_{i}(\psi ):=\frac{(1-\psi )z_{i}^{2}}{1-\psi +\psi s_{i}^{2}}.$$

The *w_i_* are not random, whereas for each value of *ψ* the $v_{i}(\psi )=w_{i}(\psi )\;z_{i}^{2}$ are random variables. The expected values of *θ*_0_*v_i_*(*ψ*_0_) for all *i* are unity.

The normalization that makes each column of *Z* sum to 0 induces one singular value of 0. It corresponds to the constant left singular vector with all entries equal to $1/\sqrt{n}$.

We use the symbol Cov to represent the empirical covariance of the elements of two n-dimensional vector arguments. Similarly, we use Var with a vector argument for the empirical variance of the elements. When the vector elements are themselves random variables the output of Cov and of Var are themselves univariate random variables.

We define *τ_k_*(*ψ*) to be a rescaled version of the empirical *k*-th central moment of the elements of the vector *w_i_*(*ψ*); that is, for *k* ≥ 2

$$\tau_{k}(\psi )=\psi^{-k}\frac{1}{n}\sum_{i=1}^{n}{\left(w_{i}(\psi )-\bar{w}\right)}^{k},\;\text{where}\;\bar{w}=\frac{1}{n}\sum_{i=1}^{n}w_{i}(\psi ).$$

We also define

$$\tau_{1}(\psi ):=\psi^{-1}\;(1-\bar{w}).$$

Note that if we define

$${\tilde{w}}_{i}(\psi ):=\psi^{-1}\;(1-w_{i}(\psi )),$$

then

$${\tilde{w}}_{i}(\psi )=\frac{s_{i}^{2}}{1-\psi +\psi s_{i}^{2}},$$

and *τ_k_*(*ψ*) is the central moment of these *w̃_i_*. Hence *τ_k_*(*ψ*) is well behaved at *ψ* = 0. It is bounded by the maximum of $s_{i}^{2k}$ and for *ψ* > 0 also by *ψ*^−^*^k^*. We write *τ_k_* with no argument for *τ_k_*(*ψ*_0_). An important quantity in the scaling of errors in our estimates will be

$$\nu :=\frac{1}{\sqrt{n\;\tau_{2}}}.$$

Since *w_i_*(*ψ*_0_) ∈ [0, 1], we know $|\tau_{k}|\;\leq 1/(2k\psi_{0}^{k})$, and $|\tau_{k+j}/\tau_{j}|\;\leq 1/(2k\psi_{0}^{k})$ for *ψ*_0_ ∈ (0, 1]. For *k* ≥ 3 we may replace 2*k* by 2*k* + 2 (*cf.* [8]). For fixed *ψ*, *w_i_*(*ψ*) is a convex function of $s_{i}^{2}$, and the mean of $s_{i}^{2}$ is unity. Jensen’s Inequality implies that *w̄* ≥ 1−*ψ*_0_ and *τ*_1_ ≤ 1. Formulas for *τ*_1_ and *τ*_2_ and *τ*_3_ under various assumptions about the singular values of the genotype matrix *Z* are derived in Section 5.

We collect our main results about the asymptotic behavior of the MLE for heritability (*ψ̂*) in Theorem 3.1 proved in Appendix A. As shown there, substituting the maximum likelihood estimator of the precision parameter *θ* into the two-parameter log likelihood leads to a one-parameter “profile log likelihood”, namely

$$(-n/2)\log\;\left(\sum w_{i}(\psi ){z_{i}}^{2}\right)+(1/2)\sum \log\;\left(w_{i}(\psi )\right)+(n/2)(\log(n)-1).$$

For each fixed realization of the random variables *z_i_*, this quantity is well-defined for any trial value of *ψ* within the open interval ( $-1/\max(s_{i}^{2}-1)$, 1). It goes to minus infinity as *ψ* becomes so negative as to approach the lower boundary, and, thanks to the singular value at zero, it also goes to minus infinity as *ψ* goes to 1. Thus the profile log likelihood has an interior maximum.

Although the true heritability parameter *ψ*_0_ is required to be non-negative, we are allowing estimated values to range below zero. We are not excluding negative estimates and we are not truncating their distribution at zero. Arguments for regarding negative estimates as meaningful within the GREML model are developed in [44]. Irrespective of those arguments, bias that would arise from truncation is already well-understood, and our focus is naturally on the more cogent question of estimator bias in the absence of truncation.

For Theorem 3.1 we construct an approximation to the maximum likelihood estimator of *ψ*_0_, expanded in powers of *ν*. The approximation is expressed in terms of *ψ*_0_, *τ*_1_, and *τ*_2_. If we think of it as a limit these quantities must be held constant or converge to their own limits as *n* → ∞. The error in the approximation is bounded in terms of higher moments of *w̃_i_*, up to *τ*_16_, so these moments must be uniformly bounded. That is, for the nonasymptotic error terms to be small these moments must all be small relative to *ν*^−1^. When *ψ*_0_ = 0 we also need the maximum of the singular values to be uniformly bounded, or, at least, to grow more slowly than any power of *n*. This condition will indeed be satisfied, with probability going to 1, in any of the cases we consider in Section 5

#### 

##### Theorem 3.1

*The maximum likelihood estimate* (*θ̂, ψ̂*) *satisfies*

(4) $$0=\text{Cov}\;\left(w(\hat{\psi }),v(\hat{\psi })\right),$$

(5) $$\hat{\theta }=\frac{1}{\bar{v}}={\left(\frac{1}{n}\sum_{i=1}^{n}v_{i}\right)}^{-1}.$$

*Furthermore, for ψ*_0_ ∈ [0, 1)

- *The MLE has negative bias on the order of ν*^2^*, i.e.* 1/*n. If we drop terms of order ν*^3^ *and higher, we get*
  (6) $$\text{Bias}=E\;\left[\hat{\psi }\right]-\psi_{0}\approx -\frac{2(1-\psi_{0})(1-\tau_{1})}{n\tau_{2}},$$
  *which is strictly negative except when ψ*_0_ = 0.
- *The MLE has variance*
  (7) $$\text{Variance}=E\;\left[{\left(\hat{\psi }-\psi_{0}\right)}^{2}\right]\approx \frac{2{\left(1-\psi_{0}\right)}^{2}}{n\;\tau_{2}}$$

*The errors are bounded by a constant times ν*^3−^*^α^ for any positive α. The constant is bounded by a universal constant for a given ψ*_0_*, but goes to* ∞ *as ψ*_0_ → 0*. At the special point ψ*_0_ = 0 *the convergence still happens in the same way as long as*

$$\lim_{n\to \infty }n^{-\alpha }\max_{1\leq i\leq n}s_{i}^{2}=0$$

*for some α* > 0.

The proof is given in Appendix A

We may identify terms in *ν* with terms in *n*^−1/2^ when it is asymptotically true that *τ*_2_ tends to a constant. In practice our expansions are more general in two regards: first, asymptotically, they should hold when *τ*_2_ decreases to zero but more slowly than 1/*n*. Then *ν*^2^ would go to zero more slowly than 1/*n*, and estimator variance would have different behavior from textbook maximum likelihood. Second, for practical purposes, our expansions appear to give serviceable approximations in simulations of cases for which *τ*_2_ is not very large compared with 1/*n*, so that *ν* is not very small, even when *n* itself is large.

One peculiarity of this situation, in comparison to textbook MLE theory, is that we are particularly concerned with the boundary case *ψ*_0_ = 0, as in the common situation where we are testing the null hypothesis {*ψ*_0_ = 0} against the alternative {*ψ*_0_ > 0}. For any finite example *ψ*_0_ = 0 is not really on the boundary of the mathematically sensible parameter range, a matter that we discuss at greater length in [44]. But asymptotically, if the large matrices (*n* → ∞) have large singular values ( $\max s_{i}^{2}\to \infty$), the parameter range shrinks down to [0, 1). Nonetheless, Theorem 3.1 guarantees that the MLE remains asymptotically unbiased and has the expected variance as long as the maximum singular value does not grow too rapidly.

The ratio of bias to standard error for *ψ̂* is

$$-\frac{\sqrt{2}(1-\tau_{1})}{\sqrt{n\tau_{2}}}.$$

The ratio will be small when *nτ*_2_ is large. It is negative, by Jensen’s inequality.

When the variance in { $s_{i}^{2}$} is small we have delta-method approximations

$$1-\tau_{1}\approx \psi_{0}(1-\psi_{0})\;\text{Var}(s_{i}^{2})$$

and

$$\tau_{2}\approx {\left(1-\psi_{0}\right)}^{2}\;\text{Var}(s_{i}^{2}).$$

The consequence is an approximate bias in *ψ̂* of −(2*ψ*_0_/*n*). These approximations may fail utterly if the eigenvalue variance is large, as it may be in stratified settings, but they correctly pick out leading terms when eigenvalue variance is small. In the null case when *ψ*_0_ = 0, the bias is zero not just to order $1/\sqrt{n}$ but actually to order 1/*n*.

For the the independent setting, we can be more precise. Exact formulas for the contributions of order 1/*n* to bias and variance in estimated heritability and for the moments of *w_i_* for the independent setting are derived in Section 5, along with expansions up to second order in *μ* = *n*/*p*. The expansions give

$$1-\tau_{1}=(1-\psi_{0})\;\psi_{0}\;\left(\mu +(2\psi_{0}^{2}-\psi_{0})\;\mu^{2}+\cdots \right)$$

and

$$\tau_{2}={\left(1-\psi_{0}\right)}^{2}\;\left(\mu +(5\psi_{0}^{2}-2\psi_{0})\mu^{2}+\cdots \right)$$

Estimator bias is given by

(8) $$\text{Bias}\;(\hat{\psi })=-\left(\frac{2\psi_{0}}{n}\right)\;\left(1+(\psi_{0}-3\psi_{0}^{2})\mu +\cdots \right)$$

Estimator variance is given by

(9) $$\text{Variance}(\hat{\psi })=\frac{2{\left(1-\psi_{0}\right)}^{2}}{n\tau_{2}}\approx \left(\frac{2}{n\;\mu }\right)\;\left(\frac{1}{1+(5\psi_{0}^{2}-2\psi_{0})\mu }\right)$$

We conclude that in the independent setting bias is indeed a very small negative number, a third-decimal effect even for samples no bigger than a thousand respondents. Variance, on the other hand, increases as the number of SNPs per person 1/*μ* increases, implying standard errors as large as 0.14 with 10, 000 people and a million SNPs. (In our independent setting, all SNPs are in linkage equilibrium.)

The contrasting “stratified setting”, as we are using the term, embraces a wide range of alternatives similar to those found in empirical cases whose genotype matrices have a subset of singular values substantially larger than singular values from the independent setting. Each genotypic singular value distribution we study represents a possible form of population structure. Since the mean of squared singular values is constrained to be unity, each large singular value must be balanced by a number of small ones. We review the behavior of bias and variance under three stylized models incorporating such balance and broadly resembling singular value distributions described in the literature. We emphasize these models all exclude confounding environmental structure, though in reality genotypic stratification almost always correlates with some degree of environmental stratification.

The first two models are built from a specification with paired point masses developed in Section 5, namely a distribution for $s_{i}^{2}$ which puts mass *β*/(*β* +1) at 1/*β* and puts mass 1/(*β* + 1) at *β*.

In the first stylized model, a moderately small proportion *α* of squared singular values are drawn from this paired-mass distribution, while the remaining 1−*α* recapitulate those from the independent setting. Means and mean squares for *w_i_* are weighted averages of expressions given in Section 5. Such a “dosage” of more widely dispersed singular values does increase *τ*_2_ and reduce the standard error of estimation, but not by much. Small *α* even in combination with large *β* limits the improvement. When *ψ* = 1/4 and *α* = 1/100 with *μ* = 1/25, standard errors drop by less than 25%. The dosage also shifts *w̄*, but the bias remains very small in comparison to the standard error for typical, sizable *n*.

In the second stylized model the cluster of singular values close to unity characteristic of the independent setting is taken out, putting *α* = 1 and leaving the paired-mass distribution on its own. For large *β*, the variance of the squared singular values is close to *β* and the moments of *w_i_* given in Section 5 lead to approximations for estimator bias and variance:

$$\text{Bias}\;(\hat{\psi })=-\left(\frac{2\;\beta }{n}\right)\;\psi_{0}\;\left[\beta {\left(1-1/\beta \right)}^{2}\psi_{0}(1-\psi_{0})+\frac{1}{\beta }\right]\approx -\left(\frac{2\;\beta }{n}\right)\;\psi_{0}^{2}\;(1-\psi_{0}),\;\text{and}\;\text{Variance}(\hat{\psi })=\left(\frac{2\;\beta }{n}\right)\;{\left[\beta {\left(1-1/\beta \right)}^{2}\psi_{0}(1-\psi_{0})+{\left(\beta -1\right)}^{-2}\right]}^{2}\approx \left(\frac{2\;\beta }{n}\right)\;\psi_{0}^{2}{\left(1-\psi_{0}\right)}^{2},$$

where the approximation holds for *ψ*_0_ > 0 and *β* large. (If *ψ*_0_ is close to 0 we need $\beta \gg \psi_{0}^{-1/3}$.) Here, the larger the variance in squared singular values (roughly *β*), the larger the variance in *ψ̂*. Instead of affecting estimator accuracy in the same fashion as *μ* = *n*/*p* (the variance of $s_{i}^{2}$ in the independent setting), *β* takes on a role like 1/*μ*, eroding accuracy as it grows. The stylized setup makes the reason apparent. Large squared singular values have to be balanced by large numbers of near-zero values to preserve the mean of unity. The transformation from *s_i_* to *w_i_* discounts the leverage of large values while the preponderance of small ones pull *w̄* toward unity and *τ*_2_ toward zero.

The third stylized model posits squared singular values from a lognormal distribution, mimicking the appearance of singular value graphs in [20] and elsewhere. Denoting the variance of the squared singular values by *γ*, under the constraint of unit mean the variance of the underlying normal is log(1 + *γ*) and its mean is −(1/2) log(1 + *γ*). Our *w_i_* then follows a so-called “logit-normal” distribution. Closed-form moments for *w_i_* are not available, but their behavior is easy to infer. As *γ* increases, squared singular values become heavily concentrated near zero and *w_i_* near 1−*ψ*. The variance of *w_i_*, that is, *τ*_2_, increases to a maximum and then falls off slowly toward zero. Moderate stratification reduces standard errors of estimation, but large departures from the independent setting raise standard errors and spoil estimates.

### 3.3. Implications of the formulas

The formulas of Section 3.2 have implications that may at first seem surprising but have logical explanations. First, in the context of Simple GREML, when we are dealing with independent random genotypes, increasing *p* — providing more data — seems to make accurate estimation harder. For fixed *n*, standard errors go up with *p*, not down. Second, stratified populations — something that would ideally be avoided in real data [51] and that the earlier analysis of [20] suggested would undermine the model even in theoretical, unconfounded data— seem up to a point to alleviate the problem of large standard error.

In fact, neither of these implications is surprising. The apparent paradox of more data producing a worse estimate dissolves when we recognise the structure of GREML. Increasing *p* does not simply provide more data. It changes the assumed set of influences on the phenotype. In Simple GREML, increasing *p* means dividing the same overall genetic effect into tinier pieces. In more complicated versions, where causal SNPs comprise a subset of (observed or unobserved) SNPs, as discussed in Section 4.4, positing larger numbers of causal SNPs similarly means dividing up the overall effect. The Law of Large Numbers tends to equalize genetic endowments among individuals, regardless of which particular SNPs happen to have the largest effects. Naturally, the situation becomes more complex when causal SNPs are sparse and linkage disequilibrium is crucial, issues that figure prominently in the literature.

It is also no surprise that some degree of population stratification, as it augments the variance of squared singular values, reduces standard errors of estimation. Zero or near-zero singular values reflect sets of individuals with high genetic similarity. Their presence allows noise to be most easily isolated from the genetic effect. It is common practice to study twins to simplify the identification of genetic effects. The reason behind the standard practice of removing relatives from a sample and pulling out population strata as fixed effects is a belief that relatives are likely to have their common genetic influences confounded with shared environment, and that genetic strata are likely to reflect stratification in non-genetic respects, hence also create confounding [36, 34, 51, 2] (though the magnitude of this effect in typical datasets is debatable [12]). The reason is not that such individuals make the statistical analysis inherently difficult.

The GREML model implies a correspondence between the covariances of the measured trait and the covariances of the high-dimensional genotype. Clearly, there is information in *Z* and **y**, but it is not immediately apparent where it is and how much is there. Passing to the diagonalization of Z^*^Z clarifies the situation: The information is in the different magnitudes of the rotated components. This is very diffuse information. We are faced with a large number of observations *z_i_* from very similar distributions, differing only in their variances, which are $\left(1-\psi +\psi s_{i}^{2})/(\theta (1-\psi )\right)$ We are trying to identify *ψ* as the parameter that best orders the *z_i_* by magnitude. It is apparent that the challenge increases as the *s_i_* become more compressed, as our formulas prove.

### 3.4. A symmetry relation

The demonstration in Section 3.2 that the GREML estimator of heritability is approximately unbiased depends on an approximation to order 1/*n* that is incomplete from a practical point of view, insofar as we do not show that terms of higher order than 1/*n* are genuinely smaller for ranges of parameters of interest.

The demonstration can be strengthened by appeal to a symmetry relation. If we replace **y** by **ỹ** = *A*^−1/2^**y**, then we obtain a sample from the same class of multivariate normal distributions defined in (2), with the matrix *A* replaced by *A*^−1^, *ψ* replaced by 1 − *ψ*, and *θ* by *θ*(1/*ψ* − 1).

A genotype matrix that would produce *A*^−1^ would not have the usual properties of the normalized genotype matrices we have been considering; in particular, such inverted GRMs may be unrealistic, in that they have low probability under typical genotypic models. Any claim that the GREML model generally yields estimates biased in one direction must depend on such properties. In principle an allowable data set that yields a positively biased heritability estimate can be paired with an alternative allowable data set (obtained by inverting the GRM) that yields a negatively biased heritability estimate, without changing the form of the model.

## 4. Model misspecification

### 4.1. Basic principles

Models that function well when fitted to data sampled from the correct distribution may produce unpredictable and unintuitive results when applied to data generated by a different albeit analogous distribution. Much of the dispute between [20] and [53] centers around the question of whether the specification of the GREML model and algorithm allows for linkage disequilibrium, and whether population stratification is adequately accounted for. [20] also considers, by means of subsampling real data, the question of whether the choice of SNPs to be investigated — as a subset of the full complement of SNPs — increases the variance of heritability estimates in ways that the standard analysis fails to capture.

The question that needs to be asked is this: Given the simplifications that we know underlie the GREML model, should we expect approximately sensible inferences to follow from approximately well specified data? We consider three types of deviation from the model:

- Shared environment;
- Measurement error;
- A small number of causal loci that are responsible for the influence of genes on phenotype, while the large majority of SNPs have no influence.

### 4.2. Shared environment

This problem is well known, and a focus of significant attention. It has long been recognized that large and small singular values of the genotype matrix are associated with potential confounding of genetic and environmental influences. Large singular values arise from stratified populations, reflecting geographical or ethnic differences that may be associated with trait differences not directly caused by the genetic differences themselves. Small singular values tend to arise from small clusters of related individuals, who are likely to be correlated in their environments as well. (In the extreme case, *C* clones produce *C* − 1 zero singular values and one singular value of size *C*.) The usual practice of working with the GREML model recognizes these problems: from the outset, population stratification has been addressed with principal component analyses and cryptic relatedness by removing samples with suspiciously high kinship [51].

We have ignored environmental effects here, focusing on situations where all assumptions of the GREML model hold exactly. The one thing we have to add to this discussion is to point out that the behavior of models such as the GREML model depends entirely on the distribution of singular values of the genotype matrix, and thus any confounding must manifest itself through these singular values. In particular, any excess variance among the singular values relative to the known limiting form in the independent setting — which models i.i.d. genotypes that, by construction, cannot be confounded — must come from latent structure between samples or between loci. Inter-sample structure almost inevitably allows the intrusion of shared environment. That is, related samples present a tradeoff: they increase spectral spread, decreasing the heritability estimator’s variance, but expose heritability estimates to potential confounding bias. Therefore, if one is convinced that the latent inter-sample structure is benign — possibly, for example, in laboratory animals or carefully controlled twin studies — the additional spectral variance improves heritability estimates.

This also explains apparent peculiarities in the distribution of squared singular values in the independent setting described in Section 5. First, it has disappointingly small variance because there is no latent sample structure. More importantly, the problem is exacerbated when *p* grows larger, as relatedness that emerges due to chance from a small number of i.i.d. draws will converge asymptotically to zero, the expected relatedness in i.i.d. data. This is something like genome-wide Mendelian randomization, and one expects these purely exogenous genotype effects to cancel out as the number of independent genotype contributions increases.

### 4.3. Measurement error

We note here that simple measurement errors do not cause any unusual problems for the mixed-effects model; as one would expect, it simply biases the estimates of heritability downward. We look here at two types of error: Independent additive error in measuring phenotypes, and independent misidentification of SNPs. Adding an independent measurement error to the phenotype simply increases the variance of the noise term *ε*, so it is equivalent to the same model with a lower value of *ϕ* (or *ψ*).

Misidentification of SNPs is slightly more complicated. Suppose that instead of observing *Z*, we observe *Ž* = *Z* + *Z̃*. We assume the entries of *Z̃* to be independent of each other, and of the noise, with expectation 0. (They obviously can’t be independent of the entries of *Z*, but *Z* is taken to be fixed, not random.) They are nonzero with probability *π*, which we assume is close to 0. Then the phenotypes will satisfy

$$y=(\overset{ˇ}{Z}-\tilde{Z})u+\varepsilon .$$

Applying the singular value decomposition *Ž* = *U* diag(*s_i_*)V^*^, we get

$$z:=U^{*}y=\text{diag}(s_{i})V^{*}u+U^{*}\left(\varepsilon -\tilde{Z}u\right).$$

The term *ε* −*Z̃***u** is approximately a vector of independent normal random variables, with mean zero and variance $\sigma_{\varepsilon }^{2}+cp\sigma_{u}^{2}\pi$, where *c* depends on the distribution of *Z̃*. Its covariance with diag(*s_i_*)V^*^u will be on the order of V^*^ Z̃, which has expectation 0 (averaged over realizations of *Z̃*), and should typically be on the order of $\sigma_{u}\sqrt{p\pi }$, meaning that the correlations are small as long as *pπ* is large.

We may conclude, then, that the model with occasional and independent misidentification of SNPs is very much like the model with increased noise in the phenotype measurement, with a downward bias in heritability estimates proportional to the error probability. While more realistic models of genotyping error may lead to different conclusions, we have assumed only that entries of *Z̄* are independent and sparse.

### 4.4. Causal loci

The usual practice of working with the GREML model recognizes that the genetic effect saturates as the number of SNPs sampled increases [27, 30, 28, 58, 11, 10]. This is generally attributed to the increasing amount of linkage to the (possibly unobserved) causal loci. Here and in the following section we analyze the effect of applying GREML in a situation where there is a small number of causal SNPs, either a small subset of the observed SNPs (Section 4.4.1) or an unobserved set that may be linked to the observed SNPs (section 4.4.2).

#### 4.4.1. Observed causal SNPs

Suppose that the genetic effect on **y** is produced by a small number *k* ≪ *n* of SNPs. Other SNPs will be linked to these, thus being indirectly correlated with **y**. We may represent this as a slightly modified version of the standard GREML model by assuming that there is a subset *η* ⊂ {1, …, *p*} of causal SNPs, and that these causal SNPs have i.i.d. normal effects, with mean 0, conditioned on the sum of their squares being a fixed number $\sigma_{g}^{2}$. We will also think of *η* as a *p*-dimensional vector with 1 in the positions corresponding to the causal SNPs and 0 elsewhere.

We write the noise variance as

$$\sigma_{e}^{2}=\frac{1-\psi_{0}}{\psi_{0}}\sigma_{g}^{2}.$$

(The “true heritability” is naturally identified with *θ* ||**u**||^2^; when all but a small number of the components of *u* are zero, this will not necessarily be very close to *ψ*_0_ unless we impose this as a condition.) When we think of the set of causal SNPs *η* as being fixed we will call this the *causal-SNP GREML* model (or CS); when we think of *η* as being a uniform randomly selected subset we call it the *random causal-SNP GREML* model (or RCS).

We wish to understand the difference between the true *ψ*_0_ and the asymptotic estimate ψ_*_ to which the estimates would converge if we had a large number of independent experiments. We define *ε* := (ψ_*_ − *ψ*_0_)/(*ψ*_0_(1 − *ψ*_0_)).

Conditioned on a fixed *η*,

$$y_{i}=\sum_{j\in \eta }Z_{ij}u_{j}+\varepsilon_{i}.$$

The MLE will converge to the closest fit (in the Kullback–Leibler sense) to the generating model. Equivalently, we seek the ψ_*_ that solves

$$E\;\left[\text{Cov}\;\left(\frac{1}{1-\psi_{*}+\psi_{*}s_{i}^{2}},\frac{z_{i}^{2}}{1-\psi_{*}+\psi_{*}s_{i}^{2}}\right)\right]=0.$$

By linearity of covariances, this becomes

(10) $$\text{Cov}\;\left(w_{i}(\psi_{*}),E[z_{i}^{2}]w_{i}(\psi_{*})\right)=0.$$

For the CS model — so, considering a fixed *η* — we define

(11) $$\gamma_{i}:=p\sum_{j\in \eta }V_{ji}^{2}-k.$$

This represents the deviation from expectation of the size of the projection of *η* onto the *i*-th right singular vector. We note for later that *γ_i_* has expectation zero (with respect to the choice of a random *η*), and is identically zero when *k* = *p*. We note as well that when *k* ≪ *p*, we will typically expect *γ_i_* + *k* to be distributed approximately like a chi-squared variable with *k* degrees of freedom.

##### Lemma 4.1

ψ_*_ satisfies

(12) $$0=\text{Cov}\;\left(w_{i}(\psi_{*}),\frac{w_{i}(\psi_{*})}{w_{i}(\psi_{0})}\right)+\frac{\psi_{0}/(1-\psi_{0})}{k\psi_{*}/(1-\psi_{*})}\left(\text{Cov}\;(w_{i}(\psi_{*}),\gamma_{i})-\text{Cov}\;(w_{i}(\psi_{*}),\gamma_{i}\;w_{i}(\psi_{*}))\right).$$

There are two ways we might use this equation. For a given choice of singular-value distribution and of true parameter *ψ*_0_, this equation defines ψ_*_ as a function of (*γ_i_*). For a given genotype matrix we could compute the distribution of the *γ_i_* jointly with the phenotypes for a random choice of possible causal sites. In this way we could more efficiently simulate the effect of restricted causality on the heritability estimates.

Alternatively, we could use the assumption that the *γ_i_* are generically i.i.d. samples from a chi-square distribution.

##### Theorem 4.2

*In the Random causal-SNP model — that is, treating the causal SNPs as a uniform random sample of all SNPs — for large n and assuming τ*_4_ ≪ *τ*_2_*,*

(13) $$E[\psi_{*}]\approx \psi_{0},$$

*and we have a relative increase in the estimation error*

(14) $$\frac{\text{Var}(\psi_{*})}{\text{Var}(\hat{\psi })}\approx \frac{\tau_{1}^{2}\psi_{0}^{2}}{k}.$$

Proofs of these results may be found in Appendix B.

Thus, the effect of a restricted set of causal sites may be assumed to be negligible — relative to the uncertainty already acknowledged in the standard analysis — as long as the phenotype is influenced by several tens of SNPs, but to increase the uncertainty substantially when fewer than 10 SNPs are involved. This effect will be exacerbated when *w̄* is small, which will be the case when the heritability is high.

To illustrate this effect, we conducted simulations in the independent setting. We took *p* = 100, 000 SNPs, and defined them to have minor allele frequencies independently chosen, uniform on [0.05, 0.5]. We then simulated genotypes for *n* =2,000 or 10,000 individuals by independently assigning a random number of minor alleles to each individual and site, according to the binomial distribution with the appropriate MAF. The genotypes corresponding to each site were then normalized to have mean 0 and variance 1. This was our genotype matrix *Z*.

We then selected a random subset of *k* sites to be causal and independently simulated 1,000 datasets using *Z*, these causal SNPs and heritability either 0.25, 0.5 or .75. The heritability was then estimated for each dataset, according to the standard MLE procedure described in Section 3. We then repeated this procedure for 100 different random choices of the causal SNPs.

To empirically estimate variance of ψ_*_, the asymptotic heritability estimate for a given set of causal SNPs, we use a standard random-effect model. Specifically, if *ψ̂_ij_* is the estimated heritability for the *j*-th simulated dataset derived from the *i*-th set of causal SNPs, we assume

$${\hat{\psi }}_{ij}=\psi_{i}^{*}+\varepsilon_{ij}$$

where the $\psi_{j}^{*}$ and the *ε_ij_* are i.i.d. Gaussian, each with an unknown variance parameter fit using lme4 [1].

**Fig 1:**
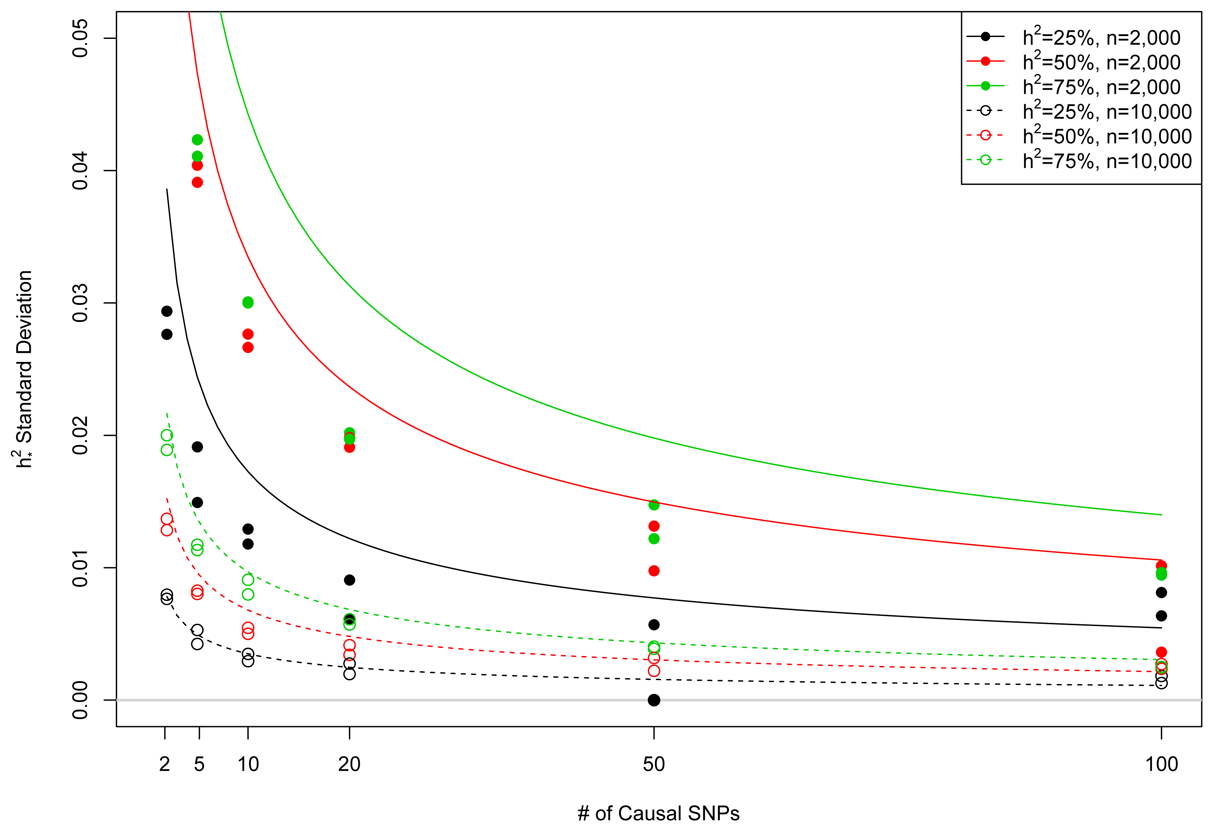
Estimated variance of average heritability estimate for 1000 random phenotypes, from each of 100 randomly selected subsets of k causal SNPs. Points give empirical variance estimates taken over simulated datasets and lines give the theoretical predictions from Theorem 4.2.

The results are summarized in Figure 1. The estimates of the variance of $\psi_{i}^{*}$ are plotted as points in Figure 1 and are compared to their theoretical predictions from (30) (continuous curves) as *k* varies. We then repeated the entire process for another, independently simulated *Z* matrix, giving two points for each combination of *k*, true *h*^2^ and *n*. Overall, the theoretical curve is fit very well, though the fit is worse when the variance between different *Z* is small (*k* large and *n* small), making it hard to separate from the much larger phenotype variance.

The code implementing this analysis is freely available online at: https://github.com/andywdahl/greml-causals

These results are consistent with previous empirical observations that randomly choosing a small number of causal SNPs inflates the variance of heritability estimates but causes no bias [56, 41, 28]. Our arguments are also in line with previous approximate characterizations of the likelihood function [22], though we approximate the profile likelihood and do not require *ψ* ≈ 0.

We go further than simulation, though, by analytically characterizing the variance inflation as a function of the number of causal SNPs. We also show the variance inflation derives from a random bias, ψ_*_ − *ψ*_0_, defined by the *γ_i_* (see Appendix C). When averaging over random *γ*, perhaps by averaging over choice of study population, *ψ̂* can be interpreted simply as an unbiased estimator with inflated variance. However, nature chooses *η* and, in real data, *V* will replicate to some degree across different datasets because of common linkage disequilibrium patterns, loosely suggesting all real analyses will partially share a common, albeit in some sense random, bias.

Theorem 4.2 is also comparable to a recent analysis of a similarly misspecified mixed model — though we have not discussed fixed effects — that showed *ψ̂* to be consistent as *n*, *p* and *k* jointly grow large [16]. However, we show (approximate) unbiasedness averaged over *η* and quantify the increase in estimator variance and its dependence on *k*. Further, we allow *u* to be non-normally distributed, which is important when modelling causal SNP effects which are known to vary over orders of magnitude.

#### 4.4.2. Unobserved causal SNPs

It is generally assumed that the SNPs that directly influence the phenotype *y_i_* are not actually among the *p* SNPs that have been measured [16], the problem of “untagged variation”. Clearly we cannot assume that the estimate of *ψ* will be unbiased. In the extreme case where the causal SNPs are independent of the observed SNPs, of course the expected estimate of *ψ* will be 0. In general we expect to see a downward bias, since the residual uncertainty about the causal SNPs will act like regression measurement error, deflating our estimate of the regression slope, which is heritability. While this has been remarked qualitatively, we are not aware of a formal derivation of the effect of untagged variation on heritability estimates.

Intuitively, it makes sense that the estimation will be as good as the best possible imputation of the causal SNPs. Of course, if we knew which were the causal SNPs we could simply include them in the sample — either measured, or the imputed values. We are assuming, though, that there is no information about the causal SNPs, which are not in the panel.

More to the point, there is no inherent meaning to “best imputation” outside the context of a particular probabilistic model generating the genotypes. For a given collection of observed and unobserved (but causal) genotype data there is an answer to the question, what is the bias in the heritability that would be estimated if we calculated the MLE from the observed genotypes? We write down this answer formally in (33), but do not see any meaningful interpretation of this formula. If we embed the genotypes in a probabilistic model, on the other hand, we are able to discuss the distribution of the unobserved bias understood as a random quantity, just as we did in Section 4.4.1.

The model is exactly the same as in Section 4.4.1, except that the *k* causal SNPs are not among the *p* observed SNPs, so the model now includes *p* + *k* SNPs in total. (We do not assume independence, so this model includes the observed-causal model as a special case, if we simply make the causal columns to be copies of some of the observed columns.)

We consider two probabilistic models:

1. The causal sites are a random sample of all sites, which are then not included in the observed genotype matrix.
2. The causal genotype matrix is generated by a linear relation
  (15) $$Z_{c}=Z_{o}B+\delta ,$$
  where *B* is a fixed *p×k* matrix, and *δ* a random *n×k* matrix with mean-zero independent entries, such that the entries in column ℓ have variance $\sigma_{\delta \ell }^{2}$. We write
  $$\sigma_{\left(\ell \right)}^{2}:=\sum_{j=1}^{p}B_{j\ell }^{2},\;\;\;\sigma_{\delta }^{2}:=\frac{1}{k}\sum_{\ell =1}^{k}\sigma_{\delta \ell }^{2}.$$
  We assume that *B* is approximately sparse — all but a small fraction of entries are negligibly small — with no more than one non-small entry in any row. That is, there is a small number of observed sites that yield nearly all the information about an individual’s causal SNPs, and these linked sites are distinct for different causal SNPs. We also assume that
  $$\sum_{\ell =1}^{k}\sigma_{\left(\ell \right)}^{2}=k-k\sigma_{\delta }^{2},$$
  which is simply a matter of ensuring that $\sigma_{g}^{2}$ is actually the additive genetic variance.

At the moment there is not much we can say further about the first model. As we will see in the discussion below, analyzing this model would require some general results relating the SVD of a matrix to the SVD of a random sample of its columns. It would be possible to investigate (33) through simulation. This would represent only a slight formalization of the simulation approach initially employed in GCTA to inflate *h*^2^ estimates *post hoc* [51].

The second model is somewhat unsatisfactory, as it produces abstract “genotypes” that are unlike the 0, 1, 2 SNP genotypes produced in real experiments. We describe it here to illustrate how the behavior of such models may be rigorously analyzed, though a more realistic version would be technically more demanding.

##### Theorem 4.3

*For large n the heritability estimates produced by the model* (15) *have a negative bias*

(16) $$\psi_{*}-\psi_{0}\approx -2\sigma_{\delta }^{2}\psi_{0}^{2}+\frac{\sigma_{\gamma }}{\tau_{2}}X$$

*for large n and small values of* $\sigma_{\delta }^{2}$*, with an error that is bounded in distribution by a uniform multiple of* $\sigma_{\delta }^{4}+\sigma_{\gamma }^{2}$*, and X is approximately standard normal (as B varies over different permutations of possible causal SNPs) and*

$$\sigma_{\gamma }^{2}=\frac{2\psi_{0}^{2}{\left(1-\psi_{0}\right)}^{2}}{nk}\;\left(\tau_{2}\tau_{1}^{2}+2\tau_{3}\tau_{1}+\tau_{4}\right)\cdot k^{-1}\sum_{\ell =1}^{k}\sigma_{\left(\ell \right)}^{4}.$$

We may draw two conclusions:

1. When *σ_δ_* is not zero (or very small) — that is, when the causal SNPs are not completely determined by the observed SNPs — there is a negative bias in the heritability estimate, proportional to $\sigma_{\delta }^{2}$, which is a measure of untagged variation.
2. When *σ_δ_* is zero this includes the situation of Section 4.4.1, if *B* is a binary matrix with only ones and zeros, so that each causal SNP is a copy of an observed SNP. The formula (16) generalizes the calculation from the previous Section, so that we see that the added uncertainty (or random bias) decreases when the information about each causal SNP is split up among multiple observed SNPs.

## 5. Singular Values

We now present formulas for moments relevant to estimator bias and variance for special cases of the distribution of squared singular values on which the GREML heritability estimates depend. We consider first the independent setting, followed by several stylized models for a stratified setting. Whereas our general treatment has only assumed a normalization of the total sum of squares of the elements of the genotype matrix, for these special cases we assume — as is usual in applications of GREML — that each column of the genotype matrix has been normalized to have unit variance as well as zero mean. The methods of this section can be extended to cases with dispersion in column variances, as well as to genotype matrices with linkage disequilibrium, but we do not pursue these extensions here.

There is a closed-form limiting expression for the empirical measure of the singular values in our independent setting as *n* grows large for fixed *μ* = *n/p*. It was discovered by Marcenko and Pastur [33] and independently by Mallows and Wachter (see [32]). We use it in the generality established by Wachter [47].

The theorems provide for almost-sure convergence to a deterministic limit. Any fixed genotype matrix *Z* will have singular values with an empirical measure close (to order 1*/n*) to this limit, if *Z* falls within a set of matrices that would have probability one in an ensemble of random matrices with independent elements. Recall that the column variances of *Z* are normalized to be close to unity.

In order to find expressions for the empirical moments across *i* of *w_i_*(*ψ*) as functions of *ψ* and *μ* = *n/p*. define the Stieltjes Transform for complex *ζ* away from the real interval [*a, b*] by

$$M(\zeta )=\mu \int_{a}^{b}\frac{dG(t)}{\zeta -t}$$

Here *t* = *s*^2^ stands for the eigenvalues corresponding to the singular values, and *dG* is the limiting empirical measure of the eigenvalues, concentrated on the interval [*a, b*] where $a={\left(1-\sqrt{n/p}\right)}^{2}$ and $b={\left(1+\sqrt{n/p}\right)}^{2}$. Conversion of the formulas for singular values to eigenvalues requires removing mass 1 − 2*μ* conventionally placed at zero and rescaling by *μ* so that *dG* has unit mass on [*a, b*].

The average of *w_i_* is given by the value of *ζM*(*ζ*)*/μ* and the average of $w_{i}^{2}$ by the value of −*ζ*_2_*M*′(*ζ*)*/μ* when we plug in *ζ* = −(1 − *ψ*)*/ψ* = −1*/ϕ*, making 1*/*(1−*ζ*) equal the heritability *ψ*. Averages of higher powers of *w_i_* are given by expressions in higher-order derivatives of *M*(*ζ*)*/μ*. Equation 2.1.1 of [47] shows that *M*(*ζ*) is the solution vanishing at infinity to the quadratic equation

$$\zeta =\frac{\mu }{M(\zeta )}+\frac{1}{1-M(\zeta )}$$

The solution can be written

$$M(\zeta )=\frac{1-\mu -\zeta -\sqrt{{\left(1-\mu -\zeta \right)}^{2}-4\mu \zeta }}{-2\zeta }$$

Here the sign on the square root is chosen to agree with the sign on 1 − *μ* − *ζ* in order to make *M*(*ζ*) vanish at infinity and to make *ζM*(*ζ*) approach *μ* at infinity.

The expression for *M*(*ζ*)*/μ* containing the square root can be differentiated in closed form, and exact expressions for the moments of *w_i_* follow from substituting 1*/*(1 − *ζ*) = *ψ*, and −*ζ* = (1 − *ψ*)*/ψ*. For practical purposes, it is helpful to expand *M*(*ζ*)*/μ* in powers of *μ*. The coefficients conveniently arrange themselves in powers of (1 − *ζ*)^−1^, streamlining differentiation and calculation of uncentered moments of any order for *w_i_*.

Specifically, for small *μ* and *ζ* either off the real axis or outside [*a, b*], we have

$$M(\zeta )/\mu \approx \frac{-1}{\left(1-\zeta \right)}-\frac{\mu }{{\left(1-\zeta \right)}^{3}}+\frac{\mu^{2}}{{\left(1-\zeta \right)}^{4}}-\frac{2\mu^{2}}{{\left(1-\zeta \right)}^{5}}\ldots$$

When 1*/*(1 − *ζ*) = *ψ*, and −*ζ* = (1 − *ψ*)*/ψ*, evaluating *ζM/μ* we find that the mean over *i* of *w_i_* is given up to second order in *μ* by

$$\bar{w}\approx (1-\psi )+(1-\psi )\psi^{2}\mu +(1-\psi )(2\psi -1)\psi^{3}\mu^{2}+\cdots ,$$

and so

$$\tau_{1}\approx 1-(1-\psi )\psi \mu -(1-\psi )(2\psi -1)\psi^{2}\mu^{2}-\cdots .$$

Differentiating *M* by *ζ* yields

$$M^{\prime }(\zeta )/\mu \approx \frac{-1}{{\left(1-\zeta \right)}^{2}}-\frac{3\mu }{{\left(1-\zeta \right)}^{4}}+\frac{4\mu^{2}}{{\left(1-\zeta \right)}^{5}}-\frac{10\mu^{2}}{{\left(1-\zeta \right)}^{6}}\cdots$$

It follows that the scaled variance over *i* of *w_i_* is given by

(17) $$\tau_{2}\approx {\left(1-\psi \right)}^{2}\mu +{\left(1-\psi \right)}^{2}(5\psi^{2}-2\psi )\mu^{2}+\cdots .$$

We also have

(18) $$\tau_{3}\approx {\left(1-\psi \right)}^{3}(1-3\psi )\mu^{2}+\cdots ,$$

(19) $$\tau_{4}\approx 2{\left(1-\psi \right)}^{4}\mu^{2}+\cdots .$$

Higher-order moments follow by successive differentiation.

Central moments of the eigenvalues themselves around their mean of unity can be found by collecting terms in powers of 1*/*(1 − *ζ*). The variance is *μ*, and to second order in *μ* the third central moment is +*μ*^2^ and the fourth central moment 2*μ*^2^. Evaluating *M*(*ζ*)*/μ* at *ζ* = 0 shows that the mean of the reciprocal eigenvalues is 1*/*(1−*μ*), and successive differentiation reveals a variance of *μ/*(1− *μ*)^3^ and a third central moment of 2*μ*^2^*/*(1 − *μ*)^5^ for the reciprocal eigenvalues.

For the stratified setting, a great variety of scenarios come under consideration. Stratified populations are expected to have genotype matrices with some or many eigenvalues substantially larger than those in the independent setting, and large eigenvalues entail large numbers of small ones, since the mean is unity.

Among the simplest stylized models for eigenvalues arising in a stratified population, one posits eigenvalues concentrated at two reciprocal values 1*/β* and *β* with weights *β/*(*β* +1) and 1*/*(*β* + 1). This two point-mass distribution, with its mean of unity, has variance (*β* −1)^2^*/*(*β* +1), close to *β* when *β* is large. Moments over *i* of *w_i_* come out to be

$$\bar{w}=\left(\frac{\beta }{\beta +1}\right)\;\left(\frac{(1-\psi )\beta }{\psi +(1-\psi )\beta }\right)+\left(\frac{1}{\beta +1}\right)\;\left(\frac{\left(1-\psi \right)}{1-\psi +\psi \beta }\right),$$

which may be simplified to

$$1-\tau_{1}=1-\frac{1-\bar{w}}{\psi }=\frac{{\left(\beta -1\right)}^{2}\psi (1-\psi )}{\beta +{\left(\beta -1\right)}^{2}\psi (1-\psi )};$$

and

$$\tau_{2}=\frac{{\left(1-\psi \right)}^{2}\beta {\left(\beta -1\right)}^{2}}{{\left[\beta +{\left(\beta -1\right)}^{2}\psi (1-\psi )\right]}^{2}}.$$

For large *β*, the mean of *w_i_* is close to 1 and the variance of *w_i_* is close to 1*/β*. In contrast to the independent setting, where the variance of *w_i_* is roughly proportional to the eigenvalue variance, in this stylized model for the stratified setting, the variance of *w_i_* is roughly inversely proportional to the eigenvalue variance. Implications for bias and variance for GREML heritability estimates have been described in Section 3.2.

## 6. Discussion

In the simple setting when assumptions are satisfied, we have shown that threats to the accuracy of GREML heritability estimates arise not from bias but from potentially large standard errors. Our findings run counter to some recent criticisms of GREML.

We have also evaluated the bias arising from fixed but unknown structured subsets for causal SNPs. This can be substantial in principle, but seems likely to be disabling in practice. We have argued that the structures that amplify bias are likely to be the exception rather than the rule. In a separate work [44] we also show how the approach presented here offers some insights into one other well-known source of bias, the presumed need to truncate negative heratibility estimates.

Standard errors for GREML estimates of heritabilities depend on the dispersion in the squared singular values of the genotype matrix. In this regard, an idealized baseline case, the independent setting, is something like a worst-case scenario. Here, drawing on eigenvalue theory, we have given explicit expansions for standard error, as well as bias, in terms of the ratio of respondents to SNPs. Implications of our formulas have been reviewed in Section 3.3. Departures from the independent setting which augment the dispersion of the squared singular values can improve the statistical properties of the estimates, but only up to a point and only at a cost in substantive interpretability. Linkage disequilibrium augments dispersion. Unexpunged population stratification augments dispersion. Their relative roles and their signatures are not yet clear. A priority for future research is analysis of empirical genotype matrices and their sets of singular values, the natural next step in elucidating the statistical properties of random effects models for genetic heritability.

## Appendix A: Proof of Theorem 3.1

We recall that

$$w_{i}(\psi )=\frac{1-\psi }{1-\psi +\psi s_{i}^{2}},\;v_{i}(\psi )=w_{i}(\psi )z_{i}^{2},\;{\tilde{w}}_{i}(\psi )=\frac{s_{i}^{2}}{1-\psi +\psi s_{i}^{2}}.$$

*τ_k_* is the k-th order central moment of *w̃_i_*(*ψ*_0_).

The log likelihood is a sum over *n* given by

$$(1/2)\sum \left(\log(w_{i})-\theta w_{i}z_{i}^{2}+\log(\theta )\right).$$

The partial derivative of the log likelihood with respect to *θ* is

(20) $$\frac{\partial \ell }{\partial \theta }=\frac{n}{2\theta }-\frac{1}{2}\sum_{i=1}^{n}\frac{(1-\psi )\;z_{i}^{2}}{1-\psi +\psi s_{i}^{2}}$$

Solving *∂*ℓ*/∂θ* = 0, we get

$$\hat{\theta }={\left(\frac{1}{n}\sum_{i=1}^{n}\frac{(1-\psi )z_{i}^{2}}{1-\psi +\psi s_{i}^{2}}\right)}^{-1}.$$

Substituting into (3) we get the profile likelihood

(21) $$\ell_{P}(\psi )=-\frac{n}{2}\log\left(\sum_{i=1}^{n}\frac{z_{i}^{2}}{1-\psi +\psi s_{i}^{2}}\right)-\frac{1}{2}\sum_{i=1}^{n}\log(1-\psi +\psi s_{i}^{2})+\frac{n}{2}(\log n-1).$$

The score function is then

(22) $$\frac{d\ell_{P}}{d\psi }=\frac{1}{2(1-\psi )}\left[{\left(n^{-1}\sum_{i=1}^{n}\frac{z_{i}^{2}}{1-\psi +\psi s_{i}^{2}}\right)}^{-1}\;\left(\sum_{i=1}^{n}\frac{z_{i}^{2}s_{i}^{2}}{{\left(1-\psi +\psi s_{i}^{2}\right)}^{2}}-\sum_{i=1}^{n}\frac{s_{i}^{2}}{1-\psi +\psi s_{i}^{2}}\right)\right]$$

(23) $$=-\frac{n}{2\psi {\left(1-\psi \right)}^{2}\bar{v}}\text{Cov}(w,v)\;=\frac{n}{2{\left(1-\psi \right)}^{2}\bar{v}}\text{Cov}(\tilde{w},v)$$

We have now arrived at our profile likelihood equation. Setting the left-hand side of equation (23) to zero, we have the maximum likelihood estimate of *ψ* equal to a root of the univariate equation

(24) 0=Cov(w(ψ),v(ψ))

The estimate is a function of the transformed observations **z** and of the singular values *s_i_* of the scaled genotype matrix $Z/\sqrt{p}$.

Our next task is to express equation (24) in terms of a power series in the differences *ψ* − *ψ*_0_. The right-hand side of equation (24) as a function of *ψ* for any fixed realization of the random quantities $z_{i}^{2}$ is a polynomial in *ψ* times a weighted sum of fractions $1/(1+\psi (s_{i}^{2}-1))$, and it is an analytic function everywhere except on the interval ( $-1/\max(s_{i}^{2}-1)$*,* 1), hence on a neighborhood of [0*,* 1).

We will use *K_j_* throughout the following discussion, for different indices *j*, to represent constants that are bounded by a universal constant times a power of *w_*_* := max{*w̃_i_*}. We will use *V_j_* to represent a random variable — a function of **v** — that has fourth moment bounded by a power of *w_*_*.

We define

$$\varepsilon :=\frac{\psi -\psi_{0}}{1-\psi_{0}}.$$

We set out to find a root of equation (24) with *ε* in the “search interval” (−2*δ_*_,* +2*δ_*_*), where

$$\delta_{*}:=\max\;\left\{\frac{0.01}{w_{*}},\frac{1}{6w_{*}^{2}\sqrt{K_{3}}}\right\},$$

and *K*_3_ is a universal constant, to be defined below. We use the relation

$$v_{i}(\psi )=v_{i}(\psi_{0})\;{\left(1+\frac{\varepsilon }{1-\varepsilon }{\tilde{w}}_{i}(\psi_{0})\right)}^{-1},\;w_{i}(\psi )=w_{i}(\psi_{0})\;{\left(1+\frac{\varepsilon }{1-\varepsilon }{\tilde{w}}_{i}(\psi_{0})\right)}^{-1}.$$

For the rest of the proof we will write, unless otherwise indicated, *w̃* for *w̃*(*ψ*_0_), **w** for **w**(*ψ*_0_), and so on. We use the third-order Taylor expansion

$${\left(1+\frac{\varepsilon }{1-\varepsilon }{\tilde{w}}_{i}\right)}^{-1}=\frac{1-\varepsilon }{1+\varepsilon ({\tilde{w}}_{i}-1)}\;=1-\varepsilon {\tilde{w}}_{i}+\varepsilon^{2}\;\left[{\tilde{w}}_{i}^{2}-{\tilde{w}}_{i}\right]-\varepsilon^{3}\;\left[{\tilde{w}}_{i}^{3}-2{\tilde{w}}_{i}^{2}+{\tilde{w}}_{i}\right]+R_{i}(\varepsilon ),$$

where the remainder term *R_i_*(*ε*) is bounded by

$$|R_{i}(\varepsilon )|\;\leq \varepsilon^{4}\;{\left({\tilde{w}}_{i}-1\right)}^{3}\;{\tilde{w}}_{i}\;{\left(1+\varepsilon (1-{\tilde{w}}_{i})\right)}^{-5}\;\leq 2w_{*}^{4}\varepsilon^{4}.$$

We apply this to expand the score equation up to third order in *ε*. (This expansion is the usual one for expressing asymptotic variance in terms of Fisher information, but we need explicit formulas for the sake of our bias approximation, and for a similar expansion in section 4.4.) For most purposes it would suffice to expand only out to second order. An extra order is required to make the estimates work consistently for *ψ*_0_ near 0, where the lowest order coefficients may vanish. For this reason, we may ignore — or, rather, lump into the final error term — any terms that are third-order or higher in *ε* and include in addition a factor of *ν* or *ψ*_0_.

By the bilinearity of covariance,

$$\text{Cov}(w(\psi ),v(\psi ))=\text{Cov}(w,v)-\varepsilon \;[\text{Cov}\;(w\tilde{w},v)+\text{Cov}\;(w,v\tilde{w})]\;+\varepsilon^{2}\;\left[\text{Cov}\;(v\tilde{w},w\tilde{w})+\text{Cov}\;\left(v,w{\tilde{w}}^{2}\right)+\text{Cov}\;\left(w,v{\tilde{w}}^{2}\right)-\text{Cov}\;(w\tilde{w},v)-\text{Cov}\;(w,v\tilde{w})\right]\;-\varepsilon^{3}\;\left[\text{Cov}\;\left(v,w\;\left({\tilde{w}}^{3}-2{\tilde{w}}^{2}+\tilde{w}\right)\right)+\text{Cov}\;\left(v\;\left({\tilde{w}}^{3}-2{\tilde{w}}^{2}+\tilde{w}\right),w\right)+\text{Cov}\;\left(\tilde{w}v,\left({\tilde{w}}^{2}-\tilde{w}\right)w\right)+\text{Cov}\;\left(({\tilde{w}}^{2}-\tilde{w})v,\tilde{w}w\right)\right]\;+\varepsilon^{4}V_{4}(\varepsilon ),$$

The factor *V*_4_ in the remainder term is a random variable, depending on the *v_i_*(*ψ*_0_), that is bounded by

$$|V_{4}(\varepsilon )|\;\leq 8w_{*}^{4}\cdot \frac{1}{n}\sum_{i=1}^{n}v_{i}(\psi_{0})$$

as *ε* ranges over our search interval.

We write *S*(*ε*) for the multiple of the score function given by the covariance we have just expanded, multiplied by *θ*_0_*/τ*_2_ :

(25) $$S(\varepsilon )=\psi_{0}\nu X+\varepsilon \;\left(\psi_{0}+\nu (\psi_{0}Y+X)\right)+\varepsilon^{2}\;\left[1+\psi_{0}\xi +\nu \left(-\psi_{0}W+X+(1+\psi_{0})Y\right)\right]+\varepsilon^{3}\;\left[\xi +1+\psi_{0}K_{3}+\nu V_{3}\right]+\varepsilon^{4}V_{4}(\varepsilon ),$$

where

$$X:=-{\left(\frac{n}{\tau_{2}}\right)}^{1/2}\theta_{0}\;\text{Cov}\;(\tilde{w},v),\;Y:={\left(\frac{n}{\tau_{2}}\right)}^{1/2}\;\left(\theta_{0}\;\text{Cov}\;\left({\tilde{w}}^{2},v\right)+\theta_{0}\;\text{Cov}\;\left(\tilde{w},v\tilde{w}\right)-\tau_{2}\right),\;W:={\left(\frac{n}{\tau_{2}}\right)}^{1/2}\;\left(\theta_{0}\;\text{Cov}\;\left({\tilde{w}}^{2},v\tilde{w}\right)+\theta_{0}\;\text{Cov}\;\left({\tilde{w}}^{3},v\right)+\theta_{0}\;\text{Cov}\;\left({\tilde{w}}^{2}v,\tilde{w}\right)-(2\tau_{3}+4\tau_{1}\tau_{2})\right)\;\xi :=-\frac{2\tau_{3}}{\tau_{2}}-4\tau_{1}+1\;K_{3}:=\frac{3\tau_{4}}{\tau_{2}}+\frac{10\tau_{1}\tau_{3}}{\tau_{2}}+10\tau_{1}^{2}-\frac{4\tau_{3}}{\tau_{2}}-\frac{8\tau_{1}}{\tau_{2}}.$$

We observe now that *θ*_0_**v**(*ψ*_0_) is a vector of i.i.d. $\chi_{1}^{2}$ random variables. It follows that *X*, *Y*, and *W* (which are close to normal random variables) have expectation values of zero and product moments

$$E\;X^{2}=2,\;E\;Y^{2}=\frac{8\tau_{4}}{\tau_{2}}+\frac{24\tau_{1}\tau_{3}}{\tau_{2}}+18\tau_{1}^{2}-6\tau_{2},\;E\;X\;Y=-\frac{4\tau_{3}}{\tau_{2}}-6\tau_{1}.$$

There is a uniform bound on the fourth moments of *V*_3_, *X*, *Y*, and *W*, bounded by a universal constant multiple of *τ*_16_.

We now express relevant probabilities and moments in terms of powers of *ν*. We are looking for a solution *ε̂* to the rescaled score equation *S*(*ε̂*) = 0. If *ν* is small, and if *ε̂* were on the order of *ν*, we could neglect the remainder term of order *ε*^3^, solve the resulting quadratic equation to obtain the proposal solution

(26) $$\varepsilon_{0}:=-\nu X+\nu^{2}\;\left[XY-\xi X^{2}\right]$$

and seek the full solution as a perturbation of order *ν*^3^ to our quadratic solution. Except on an exceptional event 𝒜 of small probability, defined below, this strategy succeeds.

Substituting into (25), we observe that all terms in *S*(*ε*_0_) cancel out that are not multiples of either *ν*^4^ or *ν*^3^*ψ*_0_. We assume that *ν* is small enough so that *|ε*_0_*| < δ_*_*, and consider *ε* = *ε*_0_ + *δ*, where *|δ| < δ_*_*. For all such *ε*

$$S(\varepsilon_{0}+\delta )-S(\varepsilon_{0})=\delta \;[\psi_{0}\;(1+\nu Y+(2\varepsilon_{0}+\delta )\;\left(-\frac{2\tau_{3}}{\tau_{2}}-4\tau_{1}+1+\nu (Y-W)\right)+\left(3\varepsilon_{0}^{2}+3\varepsilon_{0}\delta +\delta^{2}\right)\;K_{3})\;+\nu X+(2\varepsilon_{0}+\delta )\;(1+\nu \;(X+Y))+\left(3\varepsilon_{0}^{2}+3\varepsilon_{0}\delta +\delta^{2}\right)\;(2\xi +\nu V_{3})]+\left({\left(\varepsilon_{0}+\delta \right)}^{4}V_{4}(\varepsilon_{0}+\delta )-\varepsilon_{0}^{4}V_{4}(\varepsilon_{0})\right).$$

Consider first the case *ψ*_0_ *>* 0. Because the random variables all have bounded moments of all orders, for any *α >* 0 the exceptional event

$$A:=\{|S(\varepsilon_{0})|\;>\nu^{3-\alpha }\psi_{0}\;\text{or}\;\nu |X|\;>0.01\;\text{or}\;\nu |Y|\;>0.01\;\text{or}\;\nu |W|\;>0.01\;\text{or}\;\nu |V_{3}|\;>0.01\;\text{or}\;\max_{\varepsilon \in (-2\delta_{*},2\delta_{*})}|V_{4}(\varepsilon )|\;>10w_{*}^{4}\}$$

has probability smaller than *K_α_ν*^3^ for all *n* (hence all *ν*), where *K_α_* as usual is bounded by a universal constant times a power of *w_*_*.

By the assumption that *|ε*_0_*|* ≤ *δ_*_* and *|δ|* ≤ *δ_*_*, and the assumption that the realization of **v** falls in 𝒜, we see that the coefficient of *δ* is bounded below by *ψ*_0_*/*2. If we set *δ*_0_ := 2*ν*^3−^*^α^* it follows that *S*(*ε*_0_ + *δ*_0_) *>* 0 and *S*(*ε*_0_ − *δ*_0_) *<* 0. We conclude that, with probability at least 1 − *K_α_ν*^3−^*^α^*, there is a solution to *S*(*ε̂*) = 0 with *|ε̂* − *ε*_0_*| <* 2*ν*^3−^*^α^*. This corresponds to a solution *ψ̂* satisfying

(27) $$\hat{\psi }-\psi_{0}=(1-\psi_{0})\;\left(-\nu X+\nu^{2}\;\left[XY-\xi X^{2}\right]\right)+O\;\left(\nu^{3-\alpha }\right).$$

This random variable has distribution independent of the matrix *Z*, except through *τ*_1_*, τ*_2_*, τ*_3_, and its mean and variance are as stated in the theorem. This convergence is uniform as long as *ν* → 0 as *n*→*∞*. As *n*→*∞* the probability of multiple zeros to the score function goes to zero, so with probability approaching 1 as *ν* → 0 the unique solution converges in probability to the solution given by (27).

If *ψ*_0_ = 0 we define the exceptional event

$$A:=\{|S(\varepsilon_{0})|\;>\nu^{4-\alpha }X^{2}\;\text{or}\;\nu |X|\;>0.01\;\text{or}\;\nu |Y|\;>0.01\;\text{or}\;\nu |W|\;>0.01\;\text{or}\;\nu |V_{3}|\;>0.01\;\text{or}\;\max_{\varepsilon \in (-2\delta_{*},2\delta_{*})}|V_{4}(\varepsilon )|\;>10w_{*}^{4}\},$$

which again has probability smaller than *K_α_ν*^3^.

We note that *|ε*_0_*|* ≥ 0.9*|νX|* on 𝒜 and we restrict consideration to *|δ| < |νX|/*4. Then we can again bound the slope of *δ* in the expression for *S*(*ε*_0_ + *δ*) − *S*(*ε*_0_) to obtain, for all *|δ| < |νX|/*4, on the event 𝒜

$$S(\varepsilon_{0}+\delta )<S(\varepsilon_{0})-|\delta X|\nu /2$$

when *δ* and *X* have opposite sign and

$$S(\varepsilon_{0}+\delta )>S(\varepsilon_{0})+\delta X\nu /2$$

when they have the same sign. Thus, if we take *δ*_0_ := 2*Xν*^3−^*^α^*, we have

$$S(\varepsilon_{0}-\delta_{0})<0<S(\varepsilon_{0}+\delta_{0}),$$

and we may complete the proof as before.

Our formulas for bias and variance to order 1*/n* follow because the convergence is also in expectation and in mean square. More precisely, since the error term in the above approximation is bounded (outside of 𝒜) by (2 ∨ *|X|*)*ν*^3−^*^α^*, we have

$$\nu^{-2}\left|E\;\left[\hat{\psi }-\psi_{0}\right]-\nu^{2}(1-\psi_{0})\;\left(E\;[XY]-\xi E\;\left[X^{2}\right]\right)\right|\leq 2\nu^{1-\alpha }+K_{\alpha }\nu ,\;\nu^{-2}\left|E\;\left[{\left(\hat{\psi }-\psi_{0}\right)}^{2}\right]-\nu^{2}{\left(1-\psi_{0}\right)}^{2}E\;\left[X^{2}\right]\right|\leq (K_{0}+K_{\alpha })\nu .$$

## Appendix B: Proof of Theorem 4.2

In the CS model (so, holding *η* fixed)

$$E\;\left[z_{i}^{2}\right]=ps_{i}^{2}E\;\left[{\left(V^{*}u\right)}_{i}^{2}\right]+\sigma_{e}^{2}\;=\frac{p}{k}s_{i}^{2}\;\left(\sum_{j\in \eta }V_{ji}^{2}\right)\sigma_{g}^{2}+\sigma_{e}^{2}\;=\frac{1}{\theta }\;\left(\left(\frac{\gamma_{i}}{k}+1\right)\;\psi_{0}s_{i}^{2}+1-\psi_{0}\right)\;=\frac{1}{\theta }\left(\frac{1-\psi_{0}}{w_{i}(\psi_{0})}+\frac{\psi_{0}}{k}\gamma_{i}s_{i}^{2}\right).$$

Substituting this into the equation (10) yields (12), and completes the proof of Lemma 4.1.

The expression on the right-hand side of (12) is a linear combination of these, hence is approximately normal. In fact, since we assume *n* is large, and the *w_i_*(*ψ_*_*) are bounded, the approximation should be extremely good. The variance of the sum is the sum of the squares of the coefficients, multiplied by the common variance 2*k*. Using the equality

$$\frac{w_{i}(\psi_{*})}{w_{i}(\psi_{0})}=1+\left(\frac{\psi_{0}}{1-\psi_{0}}-\frac{\psi_{*}}{1-\psi_{*}}\right)\;s_{i}^{2}w_{i}(\psi_{*})\;=\frac{\psi_{0}/(1-\psi_{0})}{\psi_{*}/(1-\psi_{*})}+\left(1-\frac{\psi_{0}/(1-\psi_{0})}{\psi_{*}/(1-\psi_{*})}\right)\;w_{i}(\psi_{*})$$

this yields

(28) $$0=(\psi_{*}-\psi_{0})\tau_{2}(\psi_{*})+\psi_{0}(1-\psi_{*})\sigma (\psi_{*})X,$$

where *X*, defined as a function of the *γ_i_*, has standard normal distribution, and

(29) $$\sigma {\left(\psi \right)}^{2}:=\frac{2}{kn}\;\left(\tau_{1}{\left(\psi \right)}^{2}\tau_{2}(\psi )-2\tau_{1}(\psi )\tau_{3}(\psi )+\tau_{4}(\psi )\right).$$

We may now perform a perturbation analysis, on the assumption that the discrepancy *ε* = *ψ_*_* −*ψ*_0_ is small. If this is true, then we can obtain a first-order approximation for *ε* by solving

$$0=\varepsilon \tau_{2}(\psi_{0})+\psi_{0}(1-\psi_{0})\sigma (\psi_{0})X+\varepsilon \psi_{0}X((1-\psi_{0})\sigma^{\prime }(\psi_{0})-\sigma (\psi_{0})),$$

leading to

$$\varepsilon \approx -\frac{\psi_{0}(1-\psi_{0})\sigma (\psi_{0})}{\tau_{2}(\psi_{0})}X+\frac{\psi_{0}^{2}\;(1-\psi_{0})}{\tau_{2}{\left(\psi_{0}\right)}^{2}}X^{2}\;\left((1-\psi_{0})\sigma (\psi_{0})\sigma^{\prime }(\psi_{0})-\sigma {\left(\psi_{0}\right)}^{2}\right).$$

Assuming *σ/τ*_2_ and *σ*′*/τ*_2_ are both small, and that *τ*_3_ and *τ*_4_ are much smaller than *τ*_2_, we have the approximation

(30) $$\text{Var}(\psi_{*})\approx \frac{2\tau_{1}{\left(\psi_{0}\right)}^{2}\psi_{0}^{2}{\left(1-\psi_{0}\right)}^{2}}{kn\tau_{2}(\psi_{0})}.$$

If we restrict attention to the independent setting, and assume that *μ* = *n/p* is very small, then we may draw on the results in Section 5 and solve the equation explicitly. We have *τ*_1_ *≈* 1 and *τ*_2_ *≈* (1 − *ψ_*_*)_2_*μ*, while *τ*_3_ and *τ*_4_ are moderate multiples of *μ*^2^. We conclude that, to first order in *n*^−1^,

(31) $$E_{n}\left[\psi_{*}\right]=\psi_{0},\;{\text{Var}}_{\eta }(\psi_{*})=\frac{2p\psi_{0}^{2}}{kn^{2}}.$$

Regardless of their exact distribution, if the eigenvalues are sufficiently concentrated around 1 that *τ_k_* ≪ *τ*_2_, then we will have a ratio of variance due to random selection of causal SNPs to the variance due to random genetic effects and genetic phenotypes (the variance considered in standard analyses, given in (9)) of

(32) $$\frac{2\tau_{1}^{2}\psi_{0}^{2}{\left(1-\psi_{0}\right)}^{2}/kn\tau_{2}}{2{\left(1-\psi_{0}\right)}^{2}/n\tau_{2}}=\frac{\tau_{1}^{2}\psi_{0}^{2}}{k}.$$

## Appendix C: Proof of Theorem 4.3

Taking *U*Σ*V^*^* now to be the singular-value decomposition of $Z_{o}/\sqrt{p}$, we compute the rotated phenotypes

$$z=U^{*}y=U^{*}Z_{c}u+\sigma_{e}\varepsilon^{\prime }.$$

where *ε′* := *U^*^ε* is another vector of i.i.d. standard normal random variables. Thus

$$E\;[z_{i}^{2}]=\sigma_{e}^{2}+\frac{\sigma_{g}^{2}}{k}E\;{\left[U^{*}Z_{c}Z_{c}^{*}U\right]}_{ii}.$$

Using (10), the estimator *ψ_*_* will satisfy

(33) $$0=\sigma_{e}^{2}\text{Cov}\;\left(w_{i}(\psi_{*}),w_{i}(\psi_{*})\right)+\frac{\sigma_{g}^{2}}{k}\text{Cov}\;\left(w_{i}(\psi_{*}),E\;{\left[U^{*}Z_{c}Z_{c}^{*}U\right]}_{ii}\;w_{i}(\psi_{*})\right).$$

We have

$$U^{*}Z_{c}Z_{c}^{*}U=U^{*}(Z_{o}B+\delta )\;(B^{*}Z_{o}^{*}+\delta^{*})U\;=U^{*}Z_{o}BB^{*}Z_{o}^{*}U+U^{*}\delta B^{*}Z_{o}^{*}U+U^{*}Z_{o}B\delta^{*}U+U^{*}\delta \delta^{*}U.$$

Since the rows of *δ* are uncorrelated with mean 0 we have 𝔼 [*δ*] = 0 and

$$E\;\left[\delta \delta^{*}\right]=I_{n}\sum_{\ell =1}^{k}\sigma_{\delta \ell }^{2}.$$

By definition, $U^{*}Z_{o}=\sqrt{p}\sum V^{*}$. Thus

(34) $$E\;{\left[U^{*}Z_{c}Z_{c}^{*}U\right]}_{ii}=s_{i}^{2}\sum_{\ell =1}^{k}{\left(\sum_{j=1}^{p}\sqrt{p}V_{ji}B_{j\ell }\right)}^{2}+\sum_{\ell =1}^{k}\sigma_{\delta \ell }^{2}\;=\left(\gamma_{i}+k-k\sigma_{\delta }^{2}\right)\;s_{i}^{2}+k\sigma_{\delta }^{2}$$

where

$$\gamma_{i}:=\sum_{\ell =1}^{k}\sigma_{\left(\ell \right)}^{2}\;(\zeta_{i\ell }^{2}-1),\;\;\;\zeta_{i\ell }=\sigma_{\left(\ell \right)}^{-1}\left(\sum_{j=1}^{p}\sqrt{p}V_{ji}B_{j\ell }\right),$$

and so

$$E\;\left[z_{i}^{2}\right]=\theta^{-1}\;\left(1-\psi_{0}+\psi_{0}\sigma_{\delta }^{2}+(\frac{\gamma_{i}}{j}+1-\sigma_{\delta }^{2})\psi_{0}s_{i}^{2}\right)\;=\theta^{-1}(1-\psi_{0})\;\left(\frac{1-\sigma_{\delta }^{2}}{w_{i}(\psi_{0})}+2\sigma_{\delta }^{2}\phi_{0}+\frac{\phi_{0}}{k}\gamma_{i}s_{i}^{2}\right).$$

Thus (10) becomes

$$0=\left(2\sigma_{\delta }^{2}\psi_{0}\psi_{*}+(1-\sigma_{\delta }^{2})(\psi_{*}-\psi_{0})\right)\;\text{Var}(w_{i}(\psi_{*}))+\frac{\psi_{*}(1-\psi_{0})}{k}\text{Cov}\;\left(w_{i}(\psi_{*}),\gamma_{i}(1-w_{i}(\psi_{*}))\right).$$

By the same sort of calculation used in the previous proofs, and making use of the fact that the *ζ_i_*_ℓ_ are approximately independent standard normal variables, we may write this as

$$0\approx \tau_{2}\left(2\sigma_{\delta }^{2}\psi_{0}^{2}+(1-\sigma_{\delta }^{2})(\psi_{*}-\psi_{0})\right)+\sigma_{\gamma }X,$$

where *X* is approximately standard normal. Solving to first order in $\sigma_{\delta }^{2}$ yields (20). The result then follows immediately, by the same sort of calculation as above.
